# Patient-reported barriers to dietary adherence in heart failure: a qualitative substudy of the Pro-HEART trial

**DOI:** 10.1093/eurjcn/zvag092

**Published:** 2026-09-08

**Authors:** Andrew T. Reyes, Angelina Nguyen, Alona D. Angosta, Rebecca Meraz, Kelly L. Wierenga, Jennifer Kawi, Lorraine S. Evangelista

**Affiliations:** 1 School of Nursing, University of Nevada, Las Vegas, Las Vegas, NV, USA; 2 Louise Herrington School of Nursing, Baylor University, 333 North Washington Avenue, Dallas, TX 75246-1754, USA; 3 Department of Research, Cizik School of Nursing at UTHealth, 6901 Bertner Avenue, Houston, TX 77030, USA; 4 Sue & Bill Gross School of Nursing, University of California, Irvine, 854 Health Sciences Road, Irvine, CA 92697-3959, USA

**Keywords:** Dietary adherence, Heart failure, Nutrition intervention, Patient experiences, Qualitative research

## Abstract

**Aims:**

Dietary interventions can improve outcomes for individuals with heart failure and cardiometabolic comorbidities, yet long-term adherence remains difficult and contributes to high attrition in nutrition trials. Understanding participants’ lived experiences is essential for designing sustainable, patient-centred interventions. This qualitative substudy of the registered Pro-HEART randomized controlled trial explored participants’ reported barriers to adherence to high-protein or standard-protein dietary prescriptions during a 12-week intensive weight-loss phase and 12-month maintenance phase.

**Methods and results:**

Twenty-four participants from the parent trial who completed the parent trial participated in semi-structured interviews conducted immediately after the intensive phase. Two researchers conducted reflexive thematic analysis using an inductive approach until thematic saturation was reached. Participants encountered several interrelated challenges, which were categorized into six key themes: (i) psychological distress, stemming from guilt and fear of failure; (ii) tracking burden from extensive weighing and logging; (iii) technological stress related to rigid or unintuitive digital tools; (iv) cultural dissonance, particularly the mismatch between prescribed meal plans and participants’ traditional food practices; (v) social pressures stemming from family dynamics and social events; and (vi) a decrease in motivation, as initial enthusiasm waned throughout the intervention.

**Conclusion:**

Participant experiences demonstrate the need for culturally inclusive, emotionally supportive, and behaviourally feasible heart failure nutrition therapies. Future nutrition trials may improve adherence and retention by addressing psychological distress, fatigue, cultural dissonance, and social pressure.

**Registration:**

ClinicalTrials.gov: NCT1423266

## Graphical Abstract

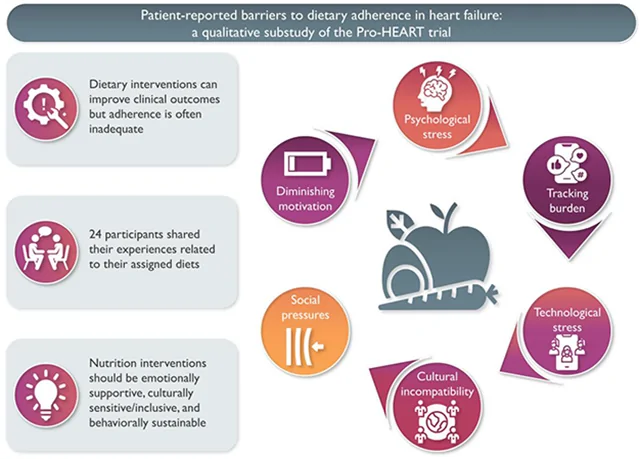

## Introduction

Numerous established benefits exist for dietary interventions in managing obesity, heart failure, type 2 diabetes mellitus, and metabolic syndrome.^1^ However, sustained adherence to these interventions remains a persistent challenge in both clinical practice and research.^2,3^ Not only are the attrition rates in dietary trials consistently high but adherence also often decreases over time among those who continue, undermining both the internal validity of clinical research and the practical application of dietary guidelines in real-world settings.^4-6^

A significant portion of the current literature emphasizes the physiological effects of dietary interventions, including weight reduction and metabolic improvements, while paying comparatively less attention to participants’ personal experiences of adhering to these regimens.^7^ While adherence rates are a common metric in quantitative research, they often fail to capture the numerous factors that contribute to low retention and engagement.^8,9^ Emotional, cultural, and social dimensions of long-term dietary change—particularly among individuals with multimorbidity—remain insufficiently understood and are rarely incorporated into intervention design.^10^

To address this gap, the present qualitative substudy examines participant-reported challenges encountered during the Pro-HEART randomized controlled trial (RCT), which compared high-protein and standard-protein dietary strategies in individuals with heart failure and either type 2 diabetes mellitus or metabolic syndrome. Participants underwent a 12-week intensive behavioural weight-loss programme followed by a 12-month maintenance phase.^11^ Using semi-structured interviews conducted at the end of the intensive phase, this study explores the emotional fatigue, cultural dissonance, social pressures, and logistical barriers that shaped participants’ adherence experiences across both dietary arms.

By identifying the specific, patient-reported reasons why individuals struggle to sustain dietary changes, this study contributes evidence needed to design culturally responsive, psychologically supportive, and behaviourally feasible nutrition programmes.^8^ These insights will be valuable for future clinical trials and public health initiatives, enhancing patient engagement and retention and improving health outcomes for individuals at risk of cardiometabolic disease.

## Methods

This qualitative substudy was embedded within the Pro-HEART trial, a previously reported randomized controlled trial designed to evaluate the effects of high-protein vs. standard-protein dietary interventions in obese individuals with heart failure and either type 2 diabetes mellitus or metabolic syndrome.^12^ The primary trial evaluated clinical outcomes related to weight reduction, metabolic markers, and health-related quality of life over 15 months, comprising a 12-week intensive weight-loss phase followed by a 12-month maintenance phase. The methodologies and findings of the parent study have been published in other works.^11^ To enhance transparency and contextualization, we added a summary of the parent trial’s design, enrolment procedures, and intervention components, consistent with COREQ reporting expectations.

### Parent trial overview

The Pro-HEART randomized controlled trial (ClinicalTrials.gov: NCT1423266) enrolled adults with obesity (BMI ≥ 30 kg/m^2^), confirmed heart failure [New York Heart Association (NYHA) class II–III], and either type 2 diabetes mellitus or metabolic syndrome. Participants were randomized to receive either a high-protein or standard-protein dietary prescription. The intervention included individualized meal plans, weekly check-ins, and structured behavioural counselling during the 12-week intensive phase, followed by monthly follow-up during the 12-month maintenance phase. Full details of the trial design, inclusion/exclusion criteria, and intervention content have been published previously.^11,12^

### Sampling and participants

This qualitative substudy examined participants’ personal experiences with the Pro-HEART dietary programme, focusing on the challenges they faced in adhering to, retaining, and sustaining behaviour change. Following the 12-week intensive phase, we used purposive sampling to recruit participants who had completed both the active and maintenance phases. All eligible participants were invited to participate in a semi-structured interview during their routine post-intervention visit. Twenty-four participants offered comprehensive accounts of their experiences with both the high- and standard-protein protocols, considering the emotional, cultural, social, and logistical factors that either aided or hindered their participation. Including only those who finished the study could introduce survivor bias, a limitation discussed in the Discussion section.

### Interview procedures

A trained qualitative researcher with experience in cardiovascular behavioural research conducted the interviews. Before starting data collection, the interviewer used reflexive journaling to record their initial assumptions. This approach helped make the analysis process more transparent.^13^ During the audio-recorded interviews, which lasted from 30 to 60 min, the researcher asked open-ended, semi-structured questions, exploring dietary adherence, motivation and retention, social and cultural challenges, tracking and monitoring experiences, psychological factors, and suggestions for programme improvement. Participants explicitly agreed to participate in the post-trial interviews and to the use of their de-identified quotations.

### Interview guide development

The investigative team developed the semi-structured interview guide based on prior literature on dietary adherence, behavioural self-monitoring, and cultural influences on nutrition.^5,8,14^ The guide was refined through pilot testing with two non-study participants to ensure clarity and relevance. To encourage detailed accounts of participants’ experiences, the questions were deliberately open-ended. The interviews were conducted immediately following the intensive phase of the intervention. The timing of this data collection was chosen to capture participants’ immediate reactions to the intervention’s most challenging aspects, thereby reducing the risk of recall bias.

### Analytic approach

The responses were analysed using thematic coding techniques to identify emerging themes and recurring patterns.^15^ The data were analysed by two independent researchers who conducted inductive coding. No new codes emerged after interviewing 24 participants, indicating data saturation. Inconsistencies were resolved through debate and consensus.^16^

We used reflexive thematic analysis following Braun and Clarke’s six-phase approach (familiarization, initial coding, theme development, review, definition, and naming).^17^ Two researchers independently coded transcripts, met regularly to compare interpretations, and maintained an audit trail documenting analytic decisions. Coding was conducted inductively to allow themes to emerge from the data rather than imposing *a priori* categories.

### Trustworthiness

Several strategies were used to strengthen trustworthiness: investigator triangulation, reflexive memoing, and maintaining an audit trail. Credibility was improved by regularly discussing findings with the research team. Dependability was ensured by carefully documenting coding choices and the different stages of analysis. Transferability was facilitated by providing comprehensive descriptions of the parent trial’s context and participants’ characteristics.^18^

### Ethical considerations

This substudy received approval from the Institutional Review Board supervising the Pro-HEART trial, and all participants provided informed consent. Furthermore, all procedures were conducted in accordance with the ethical standards of qualitative research, including confidentiality, voluntary participation, and secure data storage.

## Results

At the 12-week follow-up visit (i.e. post-intervention), the 24 participants (mean age 60.1 ± 8.2 years) were predominantly male (63%) and married (71%), with just under half (46%) having a high school education or less. The racial composition was 71% White, 17% Black, and 13% other. Functional status was evenly distributed between the NYHA classification of heart failure, class II (58%) and class III (42%). On average, participants achieved a VO_2_ max of 18.2 ± 3.9 mL· kg ^1^ · min ^1^, walked 380 ± 60 m in 6 min, and weighed 90.4 ± 12.3 kg. Baseline characteristics are presented to contextualize the lived experiences reported in the interviews.

The qualitative analysis of participant interviews identified six primary themes—psychological stress, tracking burden, technological stress, cultural incompatibility, social pressures, and diminishing motivation—that affected participants’ adherence to the dietary intervention and their overall experience in the Pro-HEART trial (See Table 1). These themes represent interconnected emotional, behavioural, cultural, and contextual elements that influenced participants’ interactions with the dietary guidelines across both phases of the trial.

### Psychological stress

Throughout the programme, a significant number of participants reported an increase in psychological stress. Specifically, many people expressed feelings of anxiety and guilt when they didn’t strictly follow the established dietary guidelines. For these individuals, even minor infractions were perceived as failures. Participants reported a sense of ‘pressure to perform’ during weigh-ins and check-ins, a sentiment that was exacerbated during emotionally challenging times, such as holidays and family events. One participant expressed, ‘The rules were so strict that one mistake felt like I had ruined everything’. This emotional burden often turned food, which is usually comforting, into a source of stress. As a result, this created a cycle of worry, self-criticism, and reduced confidence in their ability to succeed.

### Tracking burden

Participants distinguished between the cognitive and emotional burden of continuous self-monitoring (tracking burden) and the stress associated with system usability limitations and technological inflexibility (technological stress). Participants expressed frustration with the lack of adaptable, user-friendly options that could simplify the process. Participants reported feeling stressed by the tool, which made the structured diet plan seem more difficult. This experience was especially true when they were travelling, eating out, or trying to remember what they had eaten. They suggested that the app could add functionality to store or remember common meals, making it easier for them to adhere to the plan. For some, technological difficulties worsened feelings of inadequacy or discouragement, particularly for those less comfortable with digital tools. The system’s rigid structure often clashed with the realities of daily life, creating additional obstacles to sustained participation.

### Technological stress

In contrast to the emotional and cognitive load of self-monitoring, technological stress reflected frustrations with the design, rigidity, and usability of digital tracking tools. The participants expressed frustration with the lack of user-friendly, adaptable options that could simplify the process. They indicated that using the tool caused stress, making the structured dietary plan more difficult, especially when travelling, eating out, or recalling past meals. The researchers proposed that the application might incorporate features for saving or retrieving commonly eaten meals, thereby facilitating adherence to the prescribed diet. Accordingly, technological hurdles often intensified feelings of inadequacy or discouragement, particularly among those with limited digital skills. The system’s inherent inflexibility frequently clashed with the realities of daily existence, thus creating additional impediments to sustained participation.

### Cultural incompatibility

Cultural differences greatly influenced individuals’ dedication to their goals. The strict dietary regulations were difficult for many, as they disrupted established eating habits and led to feelings of loneliness and dissatisfaction. Some participants completely overhauled their usual eating routines, which resulted in less-than-ideal outcomes. Others felt frustrated because traditional cuisines, such as Caribbean and Indian dishes, were absent from the meal plans, making the diet seem unusual and disconnected from their daily experiences. Participants described a sense of cultural displacement, noting that prescribed foods did not align with their identities, family traditions, or preferred flavours. As a result, this mismatch reduced satisfaction and made the diet seem ‘foreign’ or ‘unsustainable’ over time.

### Social pressures

Participants reported that their social environments often made it harder to stick to their dietary goals. They expressed feeling excluded at gatherings, such as being unable to participate in shared meals and facing resistance from family members who were unwilling to adjust to their eating habits. Participants described feeling disconnected from their social and family environments, which caused stress, conflict, and feelings of isolation. As a result, participants reported feeling pressured to change their eating habits and their social interactions. Several participants reported that preparing different meals for their families increased their workload and emotional distress. Some individuals choose to dodge social gatherings, hoping to escape potential temptations or negative judgment. Unfortunately, this strategy often reinforced their sense of isolation.

### Diminished motivation

The interviews demonstrated a consistent decline in motivation. Interviewees frequently expressed discouragement when they encountered weight-loss plateaus or fatigue from their exercise regimens. Furthermore, some participants expressed concerns about their progress and considered ceasing their efforts due to physical and mental exhaustion. These occurrences exemplify the heightened tension that arises when attempting behavioural modification without sufficient support or adaptability. Participants characterized the initial weeks as exhilarating. Although their initial enthusiasm was evident, it diminished as the programme advanced and the demands increased. Without consistent support or flexibility, many struggled to sustain the initial drive.

The six themes identified underscore the intricate and varied challenges participants faced in the Pro-HEART dietary intervention. Specifically, the structured nature of the dietary programme, which necessitated monitoring food intake, measuring portions, and maintaining daily meal records, engendered psychological stress and burdens associated with tracking. These difficulties intersected with frustrations stemming from technological issues, cultural disparities, and social limitations. As time passed, these interconnected challenges grew increasingly intricate and arduous, affecting daily routines and diminishing motivation and engagement. Subsequently, these observations underscore the interplay of emotional, cultural, and contextual factors in shaping adherence to dietary plans and highlight the need for more flexible, culturally sensitive, and supportive dietary changes.

## Discussion

This qualitative substudy corroborates and enriches existing research on barriers to dietary adherence in persons with cardiometabolic diseases. Consistent with other research,^8^ participants in the Pro-HEART trial demonstrated a complex interplay of psychological, behavioural, and contextual factors that influenced their compliance with dietary guidelines. Prior research has shown that decreased motivation over time, emotional fatigue, and challenges with self-monitoring are key contributors to attrition in lifestyle programmes.^5,6,19^ The present findings reveal analogous themes, as individuals articulate the stress of continuous monitoring and the emotional toll of adhering to strict dietary requirements over an extended period.^14^ Participants’ descriptions of guilt, pressure, and fear of failure align with evidence that rigid dietary prescriptions can evoke negative affect and undermine self-efficacy. The embarrassment and anxiety surrounding minor dietary violations support earlier research, which shows that overly strict dietary recommendations may unintentionally evoke negative feelings and prevent engagement.^8^

This work corroborates existing knowledge and offers novel insights, particularly regarding the social and cultural determinants of adherence, which have been inadequately examined in formal clinical trials.^3,4^ Public health initiatives promote cultural adaptation. However, this approach is infrequently implemented in clinical nutrition trials, particularly for individuals with cardiovascular disease.^1^ The qualitative findings suggest that the absence of culturally relevant options may diminish individuals’ happiness and reduce their commitment to their goals and ambitions. They may also induce a sense of identity loss and isolation, a phenomenon that is seldom quantified in outcome evaluations.^20^ Participants’ accounts of cultural mismatch reinforce emerging evidence that culturally discordant dietary prescriptions can reduce acceptability, satisfaction, and long-term adherence.

Certain conclusions were self-evident. The waning desire reported by participants during the maintenance phase aligns with established behaviour change models, which predict a decline in adherence after the loss of early novelty and structured support.^20^ Similarly, the cognitive burden of meal tracking and portion control has long been recognized as a practical impediment, particularly for individuals with competing life responsibilities.^5^ However, some other outcomes were less conclusive. Several participants reported that dietary tracking generated tension and adversely affected their emotions, thereby contradicting the primary aim of behaviour management.^21^ Nevertheless, individuals often perceive that health monitoring will augment their sense of control and responsibility.^14^ These findings highlight a paradox: while self-monitoring is intended to promote awareness and accountability, its cognitive and emotional demands may overwhelm participants, particularly those managing multiple chronic conditions.

Social support is often perceived as a means of helping individuals achieve their goals; however, this investigation revealed that social circumstances frequently led to feelings of exclusion, temptation, or discomfort.^22^ The expectation that participants could navigate these contexts without assistance from a peer or individual may have further exacerbated their feelings of isolation.^6^ This underscores the importance of integrating family- or community-based strategies into nutrition interventions, particularly for populations whose eating patterns are deeply embedded in social routines.

The results provide patient-centred evidence that dietary modifications may be difficult to sustain without attention to emotional, cultural, and contextual needs, highlighting a substantial gap in the research. Conversely, extensive research has been conducted on outcome indicators, including metabolic markers, weight-loss rates, and programme adherence.^7^ There is limited interest in understanding individuals’ experiences, particularly those who may conceal their emotions or express concerns about their progress.^10^ This study demonstrates that interventions emphasizing emotional resilience, cultural context, and consistent eating behaviours are more effective than those that rely solely on dietary supplements.^23,24^ This finding underscores the significance of developing nutritional therapies that are flexible, culturally responsive, and psychologically supportive.

It is important to recognize the limitations of this study. The findings are limited by the small sample and the characteristics of the single-site parent trial from which the data were collected. The small sample size may not fully reflect the wide range of experiences of all participants.^25^ The participants who completed the study may not have accurately reported the most significant barriers to adherence, as their perspectives may differ from those of the individuals who withdrew from the study.^26,27^ We acknowledge that recall bias and social desirability bias likely contributed to the shaping of the interview responses. Social desirability bias may have influenced the reporting of issues, particularly in comments provided directly to study personnel.^8,28^ However, incorporating qualitative interviews within a rigorously designed randomized controlled trial enhances the study’s applicability and relevance in real-world contexts. Furthermore, thematic saturation was achieved, and the direct communication between participants and researchers enhances the clarity and credibility of the results, despite the limited sample size.^25^

The implications for practice and future research are significant. Nutrition programmes, particularly for individuals with chronic illnesses, must abandon uniform models in favour of adaptable approaches that are culturally specific and incorporate psychological support.^26,27^ Integrating behavioural coaching, culturally diverse meal planning, and universally applicable tracking systems may address certain identified issues.^29,30^ Providing personalized, ongoing support is essential for maintaining motivation for dietary adherence.

Future research and practice have significant implications for the field. It is crucial to transition from one-size-fits-all nutrition programmes for individuals with long-term disorders to more culturally appropriate and adaptable approaches that incorporate psychological support.^30,31^ Addressing some of the identified issues may be achieved by incorporating culturally diverse meal planning, behavioural guidance, and accessible monitoring mechanisms for all individuals.^32^ Clinical research would benefit from including real-time qualitative assessments to monitor participants’ emotional states, rather than relying solely on retrospective evaluations.^33^ Embedding ongoing qualitative feedback loops within trials may help identify emerging barriers early and support adaptive intervention strategies.

To improve the health of individuals with cardiometabolic disorders and encourage adherence to their treatment plans, it is essential to address the complex, interconnected issues identified by our research. For our upcoming Pro-HEART maintenance cohort, we will test a culturally tailored meal library alongside a simplified checklist.

## Conclusion

Individuals with heart failure and metabolic issues have difficulty adhering to specific dietary guidelines. This qualitative substudy of the Pro-HEART trial improves our understanding of these difficulties. The paper outlines six major themes that highlight the ongoing issues of high attrition and low retention in nutrition research and practice: psychological stress, tracking load, cultural discordance, social constraints, and waning motivation. Together, these findings illustrate why long-term adherence remains difficult despite structured guidance and clinical support.

Supporting individuals with heart failure requires more than providing calorie targets or nutrition education. Effective dietary interventions must account for participants’ lived realities, including the emotional strain of strict monitoring, the cultural relevance of meal options, and the social contexts in which eating occurs.^34^ Integrating culturally meaningful meal planning, improving the usability of tracking tools, and embedding behavioural and emotional support during the intervention all contribute to a greater relationship between clinical design and real-world adherence.^8^

Ultimately, these findings underscore the importance of participant perspectives in both research and clinical nutrition practice. People-centred, culturally responsive, and psychologically supportive strategies are essential for improving adherence, reducing attrition, and enhancing the long-term success of dietary interventions for individuals with cardiometabolic disease.

## Figures and Tables

**Table 1:** Six interconnected themes affecting participant experiences

Theme	Description	Representative Participant quotes
Psychological Stress	Participants reported emotional strain from strict rules, guilt when deviating, and pressure from weigh-ins or holidays. The diet shifted food from a source of comfort to one of anxiety.	*‘The rules were so strict, it felt like I couldn’t enjoy food anymore.’* ‘*It was stressful—like walking a tightrope every day.’*
Tracking Burden	Weighing, logging, and portion tracking became unsustainable and mentally draining. While feasible initially, it eventually felt unnatural, excessive, or like an added workload over time.	*‘I hated weighing my food. It felt unnatural and hard to keep up with.’* ‘*It felt like a second job—measuring, writing, calculating.’*
Technological Stress	Digital tracking tools and rigid systems heightened frustration. Participants wanted more adaptable, user-friendly tools that could ease rather than add to the burden.	*‘More flexible tracking tools—maybe an app that remembers my meals.’* ‘*It was too much, especially when traveling or eating out.’*
Cultural Incompatibility	The programme’s limited food variety and lack of cultural inclusivity left participants disconnected from their traditions and families. Substitutions often failed to capture taste, leading to dissatisfaction and isolation.	*‘I missed rice and beans. Everything was geared toward Western-style meals.’* ‘*Not at all—there weren’t any Indian food options.’*
Social Pressures	Participants struggled with family meals, social gatherings, and peer expectations. Dietary restrictions created feelings of exclusion, conflict, and stress in social environments.	*‘I stopped going to birthday parties because I couldn’t eat anything there.’* ‘*My family didn’t want to change what they ate, so I was always cooking twice.’*
Diminishing Motivation	Initial excitement waned over time. Weight loss plateaus, physical fatigue, and emotional exhaustion eroded persistence, leading some to consider abandoning their efforts.	*‘Yes, especially after the first few months when the excitement wore off.’* ‘*Toward the end, I was just tired—physically and mentally.’*

## Data Availability

The qualitative interview data supporting the findings of this study are not publicly available due to ethical and privacy considerations, as the data contain information that could compromise participant confidentiality. De-identified excerpts relevant to the study findings are included within the manuscript. Additional information about the analytic procedures may be made available from the corresponding author upon reasonable request and subject to institutional review board approval.

## Novelty

- Identification of patient-centred barriers — A qualitative substudy identified six interconnected, patient-reported barriers (psychological stress, tracking burden, technological stress, cultural incompatibility, social pressures, and diminishing motivation) that explain why long-term nutrition trials fail in terms of dietary adherence and retention.
- Cultural mismatch as a modifiable obstacle—The lack of culturally relevant meal selections was a clear, actionable barrier, demonstrating that culturally designed meal libraries can directly increase acceptance and sustained adherence.
- Monitoring tracking fatigue impacts sustainability—Excessive weighing, extensive documentation, and strict tracking methods can lead to cognitive and emotional strain, requiring simplified or adaptive monitoring.
- Participants preferred flexible, user-friendly digital tools (e.g. saved/common-meal functionality) over restrictive apps, emphasizing design principles for digital self-monitoring in clinical trials and practice.
- Social context affects adherence—Family and societal circumstances may cause isolation, temptation, or conflict. Interventions that involve family participation or social techniques can reduce these pressures.
- Emotional support keeps motivation—Decreased adherence was caused by psychological anguish, shame over lapses, and diminishing motivation, supporting behavioural coaching and emotional resilience diet therapies.
- Clinical practice and study design implications: Incorporating real-time qualitative checks, culturally adaptive protocols, and streamlined monitoring into trials and routine care can enhance the nutritional therapies for heart failure patients’ retention, external validity, and translational impact.
